# Editorial: Methods in food chemistry and food science technology

**DOI:** 10.3389/fnut.2023.1197880

**Published:** 2023-05-04

**Authors:** Blanca Hernández-Ledesma, Pilar Gómez-Cortés

**Affiliations:** Department of Bioactivity and Food Analysis, Institute of Food Science Research (CIAL, CSIC-UAM, CEI-UAM + CSIC), Madrid, Spain

**Keywords:** alternative proteins, bioactive peptides, protein allergenicity, protein quantification, lipid extraction, soft tribology, sensory quality, uridine

This Research Topic is aimed to highlight the latest experimental techniques and methods used to investigate fundamental questions in Food Chemistry/Food Science Technology research. This Topic includes technologies and up-to-date methods which help advance science. It highlights recent advances in food chemistry and improves the knowledge of bioactive components of food from a nutritional and sensorial point of view. In this special e-collection, 10 papers covering the above-mentioned aspects have been included.

By 2050, it has been estimated that the world's population will exceed 10 billion people, which will lead to a deterioration in the global food security. To avoid aggravating this problem, dietary changes to reduce the intake of animal calories and increase consumption of sustainable, nutrient-rich, and calorie-efficient products have been recommended. The nutrient-dense grain legumes are one of the most promising alternative proteins which could exert a key role in preventing global food security and environmental sustainability. Among legumes, lupin seeds have an exceptional nutritional profile, with a high protein and dietary fiber content, making them an ideal candidate to help meet the world's growing demand for supplemental sources of protein. The major storage proteins of lupin seeds, termed α-, β-, δ- and γ-conglutins, exert both nutritional and anti-nutritional properties, thus variations in the abundance of these protein families may influence the nutritional and physiological properties of different lupin varieties. Therefore, the study by Tahmasian et al. aimed to explore the conglutin protein profiles of 46 domestic and wild narrow-leaved lupin genotypes. These authors applied discovery proteomics to identify 16 known conglutin subfamilies and high-performance liquid chromatography–multiple reaction monitoring-mass spectrometry (HPLC–MRM-MS) to evaluate their abundance in the different genotypes. These authors reported large variability in the β- and δ-conglutin content among lupin varieties and identified potential hypoallergenic genotypes in several domestic cultivars. These findings will allow the development of lupin varieties with higher levels of bioactive proteins and lower allergen content, which will be of great relevance to lupin-allergic consumers and food industries.

Purple speckled kidney bean, commonly known as *Phaseolus vulgaris* L., is one of the most important edible legumes because of its high content of proteins, complex carbohydrates, and essential vitamins and minerals. In addition of its nutritional role, proteins from this legume play an important role as source of bioactive peptides. These peptides are inactive within the source protein but can exert biological activities once released by enzymatic hydrolysis, gastrointestinal digestion and/or fermentative processes. In the study of Li et al., a protein hydrolyzate of purple speckled kidney bean with pepsin and trypsin was used as the raw material to isolate, by using a macroporous resin under optimized conditions, and to purify, by reversed-phase high-performance liquid chromatography (RP-HPLC), three radical scavenging peptides which sequences were FLVDRI, FLVAPDD, and KDRVISEL. The high specificity and biocompatibility of bioactive peptides released from plant, animal, and marine protein sources make them a suitable alternative to pharmacological therapies for promoting health and treating chronic diseases of high incidence and mortality in our society. Although their promising role as food preservatives, ingredients for functional foods and nutraceuticals has been already recognized, application of bioactive peptides in the clinical and food sectors is still very limited due to some disadvantages such as toxicity, bitterness, instability, and susceptibility to enzymatic degradation in the gastrointestinal tract. Thus, it is needed to employ different strategies to enhance the bioactivity and bioavailability of bioactive peptides, and to increase the production yield from natural sources. The review by Majura et al. focuses on the existing evidence on bioactive peptides and the strategies that have been implemented to overcome their limitations and to promote the development and commercialization of peptides-based ingredients with practical applications.

Despite their high nutritional value, their key role as source of bioactive peptides, and their techno-functional and rheological properties, some food proteins can contain immunogenic peptides responsible for allergenic response in humans. Thus, gluten is a complex mixture of water insoluble proteins rich in glutamine and proline, and markedly resistant to the action of gastrointestinal enzymes. This resistance favors the release in the gut lumen of large gluten proteins-derived peptides with potential to activate inflammatory responses in celiac disease patients. The review of Mamone et al. shows the state of art of analytical and functional methods currently used to evaluate the immunogenicity potential of gluten proteins from different cereal sources, raw seed flours and complex food products. The analytical design for assessing the content and profile of gluten-immunogenic peptides is based on the harmonized oral-gastrointestinal INFOGEST protocol followed by extensive characterization of residual gluten peptides by proteomic (coupled to tandem mass spectrometry, HPLC-MS/MS) and immunochemical ELISA analyses. The functional studies described in the review are based on the evaluation of the immunostimulatory ability of gluten peptides on gut mucosa T cells or peripheral blood cells collected from celiac disease patients after being subjected to a short oral gluten challenge.

In addition to their high content of essential amino acids, mung bean proteins have been reported to exert many health benefits, such as enhancing glycolipid metabolism, preventing and managing non-alcoholic fatty liver disease, and modulating activity of antioxidant enzymes. These proteins are commonly used for producing protein beverages, and emulsion stabilizers. However, their use in staple foods such as noodles is limited because of the absence of gluten. The addition of other legume proteins, such as peas and soybean proteins, to noodles significantly enhances their taste, palatability, and cooking characteristics. Thus, to broaden the use of mung bean proteins in noodles, Diao et al. applied a hydrothermal modification method based on gel property indicators such as gel strength, water absorption capacity, and disulfide bond content to improve the gel properties of these proteins. The efficacy of the treatment was assayed adding the modified proteins to noodles, thus demonstrating their positive effects on the wheat dough properties and noodle quality. These results support the incorporation of new proteins into staple foods.

Tribology is the science of the interaction of moving surfaces, considering the lubrication and friction of the materials. In recent years, this discipline has been applied to mimic the friction between foods and the oral cavity to understand the sensory attributes of selected food products. In the exhaustive review of Corvera-Paredes et al., the authors showed the application of soft tribology to dairy products perception and explained the important role of saliva in oral processing and interaction with dairy proteins. Sensory attributes such as astringency, smoothness and creaminess were explained based on tribology experiments and it was observed that milk proteins would produce a sensory perception of astringency by salivary interaction. In contrast, dairy fat would diminish the sensation of astringency in the oral cavity, reducing the coefficient of friction and increasing the smoothness and creaminess of whole dairy products. The study of Tian et al. also highlighted the importance of composition on the sensory quality of steamed breads. These authors evaluated three varieties of rice (long-grained rice, polished round-grained rice and black rice) mixed with different flours, and determined the effect that protein, fat, several microelements and total dietary fiber exerted on steamed bread quality. The addition of rice flour significantly increased the arginine content of the mixtures and, when the amount of rice flour exceeded 15%, the elasticity and sensory score of steamed breads were gradually reduced. Although the acceptability of this type of dough needs further investigation, this work is an excellent starting point to use rice flour as a promising ingredient to improve the nutritional quality of foods, and the results would be of interest to the steamed bread and noodle industry.

On the other hand, the current Research Topic also addresses the need for simple, fast and cost-effective methods to quantitatively determine food composition. In the study of Hueso et al., the most common methods used for total protein quantification were compared in milk samples of different species and ultrafiltration products. The ultrafiltration process is widely used in the dairy industry to concentrate whey proteins, resulting in a high total solids retentate and a permeate, and its monitoring by quantifying total protein content is critical. In this research, the Kjeldahl reference method for total protein determination, which is time-consuming and requires specific instrumentation, was compared to the bicinchoninic acid assay (BCA), the detergent compatible Bradford assay and the Dumas method. It was observed that the BCA assay was significantly affected by the composition of the food matrix and its use for protein quantification in dairy samples should be avoided. In contrast, the Bradford assay and the Dumas method provided accurate results. Taking into consideration that the Dumas method is destructive and requires specific instrumentation, these authors recommend the Bradford assay to determine the total protein content in milk and ultrafiltration products. This assay provided fast and accurate results, with a small amount of sample and without the need for specific equipment, and was not influenced by the amount of non-protein nitrogen in the food sample.

Another interesting improvement of the methodology commonly used for fat extraction is the one proposed by Watanabe et al. in dairy samples. These authors found that lipid recovery from liquid infant formulas and human breast milks was lower than expected when the Röse-Gottlieb reference method was applied. The yield of neutral lipids was not improved neither by increasing the time and temperature of sample heating with ammonia water before solvent extraction, nor by increasing the volume of extraction solvent per volume of sample, or by using chloroform or dichloromethane as extraction solvents. In this study, the use of solid phase extraction cartridges with silica gels successfully improved the extraction yield of neutral lipids (~10%) in both liquid infant formula and ruminant milk. It was suggested that silica gel may help to disrupt the emulsion of the samples and improve lipid recovery. Furthermore, this approach was even more effective in extracting triglycerides from human milk, where lipid recovery was increased by >25%.

Finally, the research of Wang et al. investigated the effects of supplementation with uridine in a sow-piglets model on reproductive performance and amino acid metabolism. Uridine is the precursor of uridine monophosphate, the most abundant nucleotide in sow milk, and it is of vital importance to maintain basic cellular functions in animal tissues. The addition of uridine in the diet of sows during their second trimester of gestation significantly reduced the average number of stillborn piglets per litter, with higher average piglet weight at birth. Besides, the free amino acids profile in sow serum, newborn piglet serum and colostrum was modified by maternal uridine supplementation. The expression of amino acids transporter and the viability of pTr2 cells was also affected. This novel study showed that the addition of uridine would alter the growth of placental tissue by increasing the viability of pTr2 cells, and the amino acid metabolism in sows and their newborn piglets.

## Author contributions

BH-L and PG-C contributed equally to the writing of the manuscript and approved the submitted version.

